# Carbazole‐Containing Polymer‐Assisted Trap Passivation and Hole‐Injection Promotion for Efficient and Stable CsCu_2_I_3_‐Based Yellow LEDs

**DOI:** 10.1002/advs.202202408

**Published:** 2022-07-03

**Authors:** Zhuangzhuang Ma, Xinzhen Ji, Meng Wang, Fei Zhang, Zibin Liu, Dongwen Yang, Mochen Jia, Xu Chen, Di Wu, Yu Zhang, Xinjian Li, Zhifeng Shi, Chongxin Shan

**Affiliations:** ^1^ Key Laboratory of Materials Physics of Ministry of Education School of Physics and Microelectronics Zhengzhou University Daxue Road 75 Zhengzhou 450052 China; ^2^ State Key Laboratory on Integrated Optoelectronics College of Electronic Science and Engineering Jilin University Qianjin Street 2699 Changchun 130012 China

**Keywords:** charge carrier injection, copper halide, defect passivation, yellow light‐emitting diodes, stability

## Abstract

Perovskite light‐emitting diodes (LEDs) are emerging light sources for next‐generation lighting and display technologies; however, their development is greatly plagued by difficulty in achieving yellow electroluminescence, environmental instability, and lead toxicity. Copper halide CsCu_2_I_3_ with intrinsic yellow emission emerges as a highly promising candidate for eco‐friendly LEDs, but the electroluminescent performance is limited by defect‐related nonradiative losses and inefficient charge transport/injection. To solve these issues, a hole‐transporting poly(9‐vinlycarbazole) (PVK)‐incorporated engineering into CsCu_2_I_3_ emitter is proposed. PVK with carbazole groups is permeated at the grain boundaries of CsCu_2_I_3_ films by interacting with the uncoordinated Cu^+^, reducing the Cu_Cs_ and Cu_I_ antisite defects to increase the radiative recombination and enhancing the hole mobility to balance the charge transport/injection, resulting in substantially enhanced device performances. Eventually, the yellow LEDs exhibit an 8.5‐fold enhancement of external quantum efficiency, and the half‐lifetime reaches 14.6 h, representing the most stable yellow LEDs based on perovskite systems reported so far.

## Graphical abstract

A polymer poly(9‐vinlycarbazole) (PVK)‐modified engineering of CsCu_2_I_3_ emitter is developed to simultaneously passivate the grain boundary defects and promote the charge carrier injection for high‐performance yellow light‐emitting diodes (LEDs). As a result, the PVK‐modified device exhibits a 8.5‐fold enhancement of external quantum efficiency and the half‐lifetime reaches 14.6 h, representing the most stable yellow LEDs based on perovskite systems reported so far.

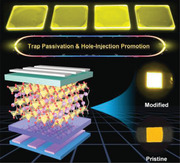

## 1 Introduction

Recently, the emerging metal‐halide perovskites have been regarded as promising candidates for the next‐generation solid‐state light‐emitting diodes (LEDs) because of their excellent optical and electrical properties including high photoluminescence quantum yield (PLQY), low trap density, superior charge‐carrier transport capability, and low‐cost solution processability.^[^ ^1^, ^2^, ^3^, ^4^, ^5^, ^6^, ^7^ ^]^ Since the first demonstration of perovskite LEDs in 2014,^[^ ^8^ ^]^ substantial progresses in this field have been witnessed through the materials composition modulation, defect passivation engineering, device structure optimization, and so on.^[^ ^9^, ^10^ ^]^ Currently, the green, red, and near‐infrared perovskite LEDs have realized admirable external quantum efficiency (EQE) surpassing 20%, marking a considerable step toward their commercial full‐color display applications.^[^ ^11^, ^12^, ^13^, ^14^, ^15^ ^]^ However, as important optoelectronic components in solid‐state lighting, flow cytometry, optogenetics, etc., the researches on the yellow‐emissive perovskite materials and devices are still scarce.^[^ ^7^ ^]^ Besides, the intrinsic toxicity of heavy metal lead and environmental instability faced by lead‐based perovskites would seriously impede their further commercial deployment.^[^ ^16^, ^17^, ^18^, ^19^ ^]^ Therefore, considering the necessity of environmental sustainability, it is a rewarding subject to fabricate yellow‐emissive LEDs based on lead‐free and stable yellow‐light emitters.

Some recent reports have explored various alternatives of Pb with less‐toxic or nontoxic elements, such as Sn(II), Ge(II), Bi(III), Sb(III), In(III), and Cu(I), and some of the resulting lead‐free substitutes have contributed to the realization of eco‐friendly perovskite LEDs.^[^ ^20^, ^21^, ^22^, ^23^, ^24^, ^25^, ^26^ ^]^ Among them, copper halide CsCu_2_I_3_ with low‐dimensional electronic structure and high environmental stability, exhibits intrinsic yellow emission, attracting considerable interests in LEDs applications.^[^ ^27^, ^28^, ^29^, ^30^ ^]^ Particularly, CsCu_2_I_3_ possesses the edge‐sharing [Cu_2_I_3_]^‐^ dimerized units separated by Cs^+^ to form a 1D chain structure and soft crystal lattice, enabling strong exction confinements and electron‒phonon coupling, resulting in an efficient self‐trapped excitons (STEs) emission.^[^ ^28^ ^]^ Besides, the weight loss temperature of CsCu_2_I_3_ is as high as 600 °C, and no significant PLQY drop occurs even after several months storage in the ambient conditions, showing an excellent luminescence stability.^[^ ^29^ ^]^ Although the synthesis and STEs emission mechanism of CsCu_2_I_3_ have been reported,^[^ ^27^ ^]^ the preparation of high‐quality films with high PLQY remains a critical challenge. Moreover, related studies on CsCu_2_I_3_‐based electroluminescence (EL) device are quite rare as far as we know. For instance, Tang et al. demonstrated a yellow LED based on the CsCu_2_I_3_ films prepared by vacuum‐deposition method, and a maximum EQE of 0.02% and a maximum luminance of ≈10 cd m^−2^ were achieved.^[^ ^29^ ^]^ By using the solution‐processed CsCu_2_I_3_ films as the emitting layer, our group fabricated an electrically‐driven LED with a broadband emission at ≈550 nm and a maximum EQE of ≈0.17%.^[^ ^30^ ^]^ Despite many efforts have been devoted, the device performances still lag far behind the conventional lead‐halide perovskites in terms of both efficiency and stability. On the one hand, the polycrystalline CsCu_2_I_3_ films prepared by the solution method usually contain a large number of crystallographic defects at grain boundaries or surfaces owing to the uncontrollable crystallization reaction.^[^ ^31^, ^32^, ^33^ ^]^ Such crystallographic defects could form inhomogeneous films morphologies, bringing charge trap states and limiting the recombination of radiative exciton.^[^ ^32^ ^]^ On the other hand, a higher requirement is put forward for the structure design of CsCu_2_I_3_‐based LED considering the relatively large bandgap (>3.6 eV) and deep valence band level (>6.0 eV) of CsCu_2_I_3_, thus it is difficult to realize matched energy band alignment and effective charge carrier injection.^[^ ^30^, ^31^ ^]^ For these reasons, exploring a feasible strategy that could suppress the defect formation and manipulate the charge carrier transport simultaneously is imperative for the continued promotion of device performances, which, to the best of our knowledge, has yet to be reported.

In this work, we developed a polymer poly(9‐vinlycarbazole) (PVK)‐modified engineering of CsCu_2_I_3_ emitter for high‐performance yellow LEDs. PVK, with carbazole group, is demonstrated to interact with the uncoordinated Cu^+^ of CsCu_2_I_3_ at the grain boundaries, passivating the Cu_Cs_ and Cu_I_ anti‐site defects and boosting the carrier radiative recombination Meanwhile, PVK modification could greatly enhance the hole injection/transport and facilitate the energy band alignment, resulting in an efficient and balanced charge carrier recombination. Benefiting from these synergistic effects, the yellow LEDs with PVK modification achieve a peak EQE of 1.35%, which is 8.5 folds that of the pristine device (0.16%). Notably, the operational stability of the modified device is highly prolonged in air ambient, showing a half‐lifetime of 14.6 h at an initial luminance of ≈100 cd m^−2^, representing the most stable yellow LEDs based on perovskite systems reported so far. These findings in this work offer a promising strategy for developing eco‐friendly and stable yellow LEDs based on copper halide semicondcutors.

## 2 Result and Discussion

**Figure 1:**
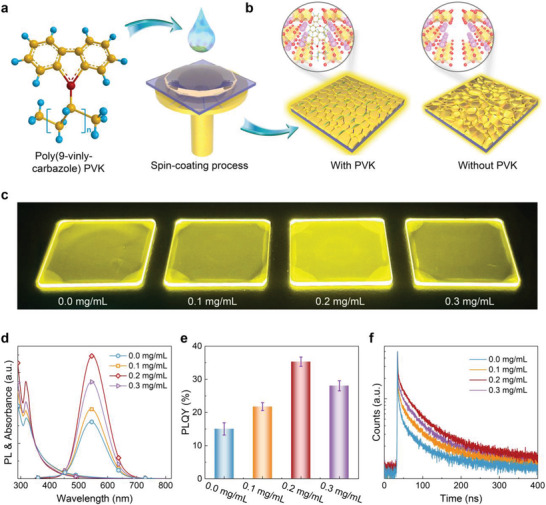
Preparation and optical properties of the CsCu_2_I_3_ films with and without PVK modification. a) Chemical structure of PVK (red, nitrogen atoms; yellow, carbon atoms; blue, hydrogen atoms). b) Schematic illustration of the spin‐coating process for synthesizing CsCu_2_I_3_ films with and without PVK modification. c) Photographs of the CsCu_2_I_3_ films prepared with different concentrations of PVK under ultraviolet light irradiation. d) Steady‐state absorption and PL spectra, e) statistical PLQY, and f) time‐resolved PL decay curves of the CsCu_2_I_3_ films prepared with different concentrations of PVK.

Due to the ionic nature of lead‐halide perovskites,^[^ ^19^ ^]^ defects are easily generated at grain boundaries or surfaces of the solution‐processed films during the rapid crystals nucleation and growth.^[^ ^20^, ^31^, ^33^ ^]^ To passivate such defects, molecular additives containing nitrogen (N) or sulfur (S) functional groups with lone electron pairs that can form intermolecular interactions with perovskites are usually used to passivate the uncoordinated ions.^[^ ^34^, ^35^, ^36^, ^37^, ^38^ ^]^ PVK is a p‐type semiconducting polymer comprising N‐based carbazole functional groups (**Figure** **1**a),^[^ ^39^, ^40^ ^]^ which are expected to not only promote the growth of CsCu_2_I_3_ films but also modulate the charge carrier transport dynamics due to the combination of lone electron pairs and good hole transport. In this experiment, PVK additives with different concentrations were introduced into the precursor solution to prepare the CsCu_2_I_3_ films by a one‐step spin‐coating method, as illustrated in Figure 1b. We first studied the influence of PVK modification on the optical properties of CsCu_2_I_3_ films by ultraviolet−visible absorption and steady‐state PL measurements. As shown in Figure 1d, four samples measured from the same set of products are all characterized by intense absorption peaks at 315 nm and broadband emission centered at 550 nm, and the corresponding Stokes shift is as large as ≈220 nm. Considering a direct and large bandgap of 4.02 eV for CsCu_2_I_3_ (Figure S1, Supporting Information), we therefore consider that the emission of CsCu_2_I_3_ originates from the STEs‐related recombination caused by the Jahn–Teller distortion of Cu tetrahedral site.^[^ ^27^, ^28^, ^30^ ^]^ The photophysical model for CsCu_2_I_3_ could be depicted using a configuration coordinate diagram, as shown in Figure S2 (Supporting Information). Notably, when the PVK concentration increases from 0 to 0.2 mg mL^−1^, the PL intensity of CsCu_2_I_3_ films was significantly enhanced, with the corresponding PLQY boosted from 15.1% to 35.5%, as presented in Figure 1e. The significantly enhanced emission of PVK‐modified samples could be visually illustrated by their photographs under ultraviolet lamp excitation, as displayed in Figure 1c. The above results suggest that the trap states in CsCu_2_I_3_ films can be effectively suppressed after PVK introduction. For further confirmation, time‐resolved PL decay measurements were carried out. As shown in Figure 1f. the PL decay curves can be fitted with a biexponential decay function, $R\left(t\right)=K_{1}\exp^{\left(-t/\tau_{1}\right)}+K_{2}\exp^{\left(-t/\tau_{2}\right)}$, where *K*_1_ and *K*_2_ are the distribution coefficients, and the fast (*τ*_1_) and slow (*τ*_2_) decay components represent the trap‐assisted recombination and radiative recombination lifetime, respectively.^[^ ^36^, ^37^ ^]^ The specific fitting parameters were summarized in Table S1 (Supporting Information). One can see that the pristine CsCu_2_I_3_ films (0 mg mL^−1^) show a smallest *τ*_1_ (1.84 ns) with the largest *K*_1_ (80.2%), reflecting severe nonradiative recombination due to the presence of a large number of defect states.^[^ ^41^, ^42^ ^]^ While, the PVK‐modified CsCu_2_I_3_ sample (0.2 mg mL^−1^) has the highest *τ*_2_ (49.85 ns) with the largest *K*_2_ (39.2%), producing a longer PL average lifetime (*τ*_ave._) of ≈21.4 ns than that of the pristine counterpart (≈7.1 ns). The prolonged *τ*_ave._ unveils an overall reduction of nonradiative recombination centers within the CsCu_2_I_3_ films, and the generated excitons are more likely to recombine with the radiative paths.^[^ ^36^ ^]^ Thus, the effcient usage of excited carriers is favorable, which is consistent with the enhanced PLQY, showing strong evidence of the reduced density of defect states after PVK modification. Such improved emission performance is a positive signal of high‐quality CsCu_2_I_3_ films, facilitating their application as an effective light emitter in LEDs.^[^ ^43^ ^]^

**Figure 2:**
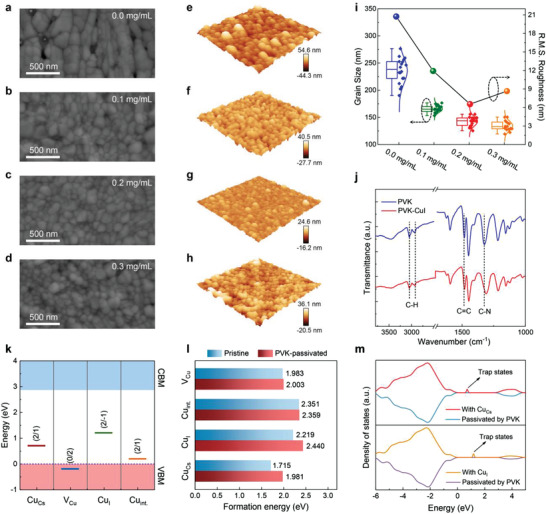
Morphologies and passivation mechanisms of the PVK‐modified CsCu_2_I_3_ films. a–d) Top‐view SEM images, and e–h) AFM images of the CsCu_2_I_3_ films prepared with different concentrations of PVK. i) Grain size distribution and R.M.S. roughness evolution of the CsCu_2_I_3_ films with different PVK concentrations. j) FTIR spectra of pure PVK and PVK‐CuI mixture. k) Calculated transition energy levels of Cu‐related defects in CsCu_2_I_3_. l) Formation energy of Cu‐related defects in pristine and PVK‐modified CsCu_2_I_3_ under Cu poor conditions. m) DOS spectra of CsCu_2_I_3_ with Cu_Cs_ defect (upper), Cu_I_ defect (bottom), and corresponding defect passivation by PVK.

Aside from the emission properties, a dense and smooth films morphology is another prerequisite for fabricating high‐efficiency LEDs.^[^ ^44^ ^]^ We therefore examined the morphological characteristics of the PVK‐modified CsCu_2_I_3_ films by performing the scanning electron microscopy (SEM) and atomic force microscopy (AFM) measurements. One can observe that the morphology evolution of CsCu_2_I_3_ films shows distinct trends after PVK introduction. As shown in **Figure** **2** **a**, the pristine CsCu_2_I_3_ films display poor morphologies with a heterogeneous appearance, a large roughness, and a high density of pinholes. With the increase of PVK concentration (Figures 2b‐d), the average grain size of CsCu_2_I_3_ films decreases monotonically from ≈253 nm (0 mg mL^−1^) to ≈130 nm (0.3 mg mL^−1^), as plotted in Figure 2i, and a well‐compact and uniform surface without any pinholes was achieved finally. The reduced grain size after PVK modification leads to an increase in the exciton binding energy of CsCu_2_I_3_ from 198.2 to 257.1 meV (Figure S3, Supporting Information), thus resulting in an enhanced radiative recombination by spatially confining excitons in the nanograins.^[^ ^44^ ^]^ Meanwhile, such nanofilms exhibit a smaller exciton diffusion length (≈106.2 nm) than previously reported bulk crystals (≈2.506 µm) (Figure S4, Supporting Information),^[^ ^27^ ^]^ suggesting a reduced possibility of excitons dissociating into free carriers without radiation, making it an excellent candidate for light emitter.^[^ ^45^ ^]^ Moreover, the Root‐Mean‐Square (R.M.S.) roughness of the optimal PVK‐modified films (0.2 mg mL^−1^) is measured to be ≈6.4 nm, which is lower than that of the pristine films (≈20.8 nm). Such a significant change confirms that the PVK modification could make the films surface smoother, as seen in Figures 2e‐i. Visibly, the improved surface coverage, decreased grain size, and reduced surface roughness of CsCu_2_I_3_ films could be attributed to the effective PVK modification, and such morphological characteristics are conducive to a reduced leakage current and an enhanced operation stability in electrically‐driven LEDs because the undesired current pathways associated with defect states were substantially suppressed.^[^ ^42^ ^]^

X‐ray diffraction (XRD) measurements were also performed to explore the effects of PVK modification on the crystallinity of CsCu_2_I_3_ films. As shown in Figure S5 (Supporting Information), the pristine and PVK‐modified samples show the analogous diffraction peaks at 10.77°, 21.64°, 26.19°, and 34.11°, representing the (110), (220), (221), and (202) crystal planes of orthorhombic CsCu_2_I_3_ (JCPDS no. 77‒0069),^[^ ^30^ ^]^ indicating no structural deterioration after the introduction of PVK. Besides, we observed that the peak intensity of (221) plane for PVK‐modified samples is relatively higher, which may result from the ordered crystallization of CsCu_2_I_3_ grains after PVK modification. Based on the above observations, we believe that the incorporated PVK interacts with the components of CsCu_2_I_3_ during the films growth. To confirm this speculation, a Fourier transform infrared (FTIR) spectroscopy study was conducted. As shown in Figure 2j, the pure PVK exhibits the characteristic peaks at 2931 cm^‐1^ (aliphatic C—H stretch from the polymer backbone), 3046 cm^‐1^ (aromatic C—H stretch), 1482 cm^‐1^ (C=C stretch), and 1324 cm^‐1^ (C—N stretch of vinyl carbazole).^[^ ^39^, ^40^ ^]^ While, after blending with CuI, the C—N characteristic peak shifts to a lower wavenumber of 1311 cm^‐1^. Such shift in FTIR peak position means the weakening or lengthening of the bonds. Considering the chemical structures of carbazole in PVK, it is expected that a chemical interaction was formed between the N atoms in PVK and Cu^+^ ions in CsCu_2_I_3_ films.^[^ ^40^ ^]^ Further, X‐ray photoelectron spectroscopy (XPS) measurements were carried out to examine the interactions. As shown in Figure S6 (Supporting Information), the characteristic peaks of Cu 2p_3/2_ and Cu 2p_1/2_ in PVK‐modified CsCu_2_I_3_ display obvious shifts to lower binding energy by more than 0.3 eV compared to the pristine sample, which can be attributed to the change in electron cloud density. Therefore, we reasonably infer that the lone electron pair of the N atom in PVK polymer is successfully delocalized through the interaction with the uncoordinated Cu^+^.

Density functional theory (DFT) calculations were further carried out to clarify the underlying mechanism of defect passivation by PVK in CsCu_2_I_3_ films.^[^ ^25^ ^]^ As mentioned above, PVK could interact with the uncoordinated Cu^+^, we thus investigated four Cu‐related defects in CsCu_2_I_3_, including two anti‐site defects (Cu_Cs_ and Cu_I_), one interstitial defect (Cu_int._), and one vacancy defect (V_Cu_). Figure 2k shows the calculated transition energy levels for considered four Cu‐related defects in CsCu_2_I_3_. One can observe that two kinds of anti‐site defects (Cu_Cs_ and Cu_I_) produce deep energy levels in the bandgap, which attract electrons/holes and act as Shockley‐Read‐Hall nonradiative recombination centers, affecting the carrier recombination dynamics and causing a degraded PLQY.^[^ ^32^ ^]^ Moreover, the defect formation energies of Cu_Cs_, Cu_I_, Cu_int._, and V_Cu_ in pristine CsCu_2_I_3_ are calculated as 1.715, 2.219, 2.351, and 1.983 eV, respectively (Figure 2l). While, once the PVK was introduced, the formation energies of Cu_Cs_ and Cu_I_ increase to 1.981 and 2.440 eV, respectively, while those of Cu_int._ and V_Cu_ are almost unchanged, suggesting that the Cu_Cs_ and Cu_I_ defects were effectively passivated (Figure 2l, Table S2, Supporting Information). The passivation mechanism of PVK is further investigated by calculating the projected density of states (DOS).^[^ ^33^ ^]^ As shown in Figure 2m, the DOS of CsCu_2_I_3_ with Cu_Cs_ (upper) and Cu_I_ defect (bottom) exhibits apparent defect states located in the bandgap, which can trap the charge carriers. While, these defect states can be effectively weakened after PVK passivation (Figure 2m), indicating that the incorporated PVK could greatly eliminate the trap states and prevent the trapping of carriers in CsCu_2_I_3_. All these results clearly reveal the defect passivation function of PVK modification.

**Figure 3:**
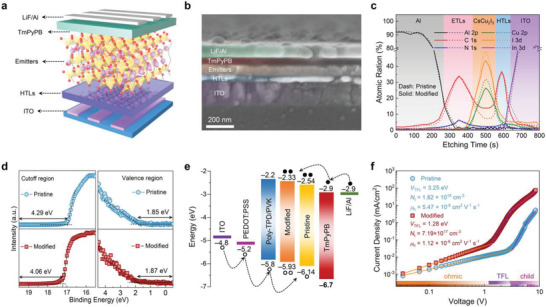
Device structure and electrical properties of CsCu_2_I_3_ films‐based LEDs. a) Schematic diagram, and b) cross‐sectional SEM image of the proposed LEDs. c) Depth‐profiling XPS analysis of the device with (solid) and without (dash) PVK modification. d) UPS spectra of the pristine and PVK‐modified (0.2 mg mL^−1^) CsCu_2_I_3_ films. e) Energy band diagram of the heterostructure device. f) Double logarithmic current density–voltage curves of the hole‐only devices based on pristine and PVK‐modified CsCu_2_I_3_ films.

Subsequently, we fabricated two yellow LEDs based on the pristine and PVK‐modified CsCu_2_I_3_ films to evaluate the effect of PVK on device performance. **Figure** **3**a shows the device architecture used in this study, which presents a multilayer heterostructure consisting of ITO/poly(3,4‐ethylenedioxythiophene):poly‐(styrenesul‐fonate) (PEDOT:PSS)/poly [bis (4‐phenyl) (4‐butylphenyl) amine] (Poly‐TPD)/PVK/CsCu_2_I_3_/1,3,5‐tri (m‐pyrid‐3‐yl‐phenyl)benzene (TmPyPB)/LiF/Al. In this device, ITO (150 nm), PEDOT:PSS/Poly‐TPD/PVK (55 nm), CsCu_2_I_3_ films (75 nm), TmPyPB (55 nm), and LiF/Al (1/100 nm) were used as anodes, hole transport layers (HTLs), emissive layer, electron transport layers (ETLs), and cathodes, respectively. The thickness of each functional layer is estimated from the cross‐sectional SEM image displayed in Figure 3b. To provide a direct and solid evidence for the location of PVK in the CsCu_2_I_3_ films, depth‐profiling XPS measurement was carried out. In this measurement, the device was sputtered with Ar ions starting from the Al layer with different cycles,^[^ ^43^ ^]^ and the atomic ratios of different elements at various sputtering cycles provide the clues to the composition distribution along the device thickness. As shown in Figure 3c, the elemental signals with a typical PVK carbon to nitrogen atomic ratio of 14:1 spread over the entire CsCu_2_I_3_ films in the PVK‐modified device,^[^ ^39^ ^]^ providing the direct evidence that PVK mainly exists inside the films. Furthermore, the atomic ratio of copper to iodine in the depth‐profiling CsCu_2_I_3_ films maintained at ≈2:3 was detected. These results confirm that PVK introduction does not affect the crystal structure of CsCu_2_I_3_, but only locates at the grain boundaries of the polycrystalline films. The transmission electron microscopy images displayed in Figure S7 (Supporting Information), convincingly verify the above descriptions, where a clear amorphous wall of PVK was observed among the adjacent CsCu_2_I_3_ grains.

To illustrate the effect of PVK incorporation on band alignment of the device, ultraviolet photoelectron spectroscopy (UPS) measurements were conducted. As depicted in Figure 3d, according to the secondary electron cutoff region of the films, the Fermi levels of pristine and PVK‐modified (0.2 mg mL^−1^) CsCu_2_I_3_ were determined to be −4.29 and −4.06 eV, respectively. Thus a significant increase in valence band maximum (VBM) from −6.14 to −5.93 eV occurs after the introduction of PVK. Note that the optical bandgap of both pristine and PVK‐modified CsCu_2_I_3_ are 3.60 eV, as shown in Figure S8 (Supporting Information), indicating almost no change in the bandgap by trace amount introduction of PVK.^[^ ^34^ ^]^ Based on these values, the conduction band minimum (CBM) of the pristine and PVK‐modified CsCu_2_I_3_ were calculated as −2.54 and −2.33 eV, respectively. Figure 3e illustrates the energy band diagram of all functional layers relative to the vacuum level. One can see that the hole injection barrier between the HTL and emitter is significantly reduced after adding PVK, which is beneficial for hole injection and supports a more balanced charge transport that enabling a high recombination efficiency of carriers in device.^[^ ^13^, ^33^, ^35^ ^]^

Further, we quantitatively explored the influence of PVK incorporation on the electrical properties (including defect density and charge carrier mobility) of CsCu_2_I_3_ by constructing the hole‐only device (ITO/PEDOT:PSS/Poly‐TPD/PVK/CsCu_2_I_3_/TCTA/MoO_3_/Au), with the architectures shown in Figure S9 (Supporting Information). Figure 3f presents the current density–voltage curves of the hole‐only device based on the pristine and PVK‐modified CsCu_2_I_3_ in the space‐charge‐limited current (SCLC) mode. The incipiently linear increase in current manifests an Ohmic response, but the curve becomes nonlinear when the bias voltage exceeds the kink point, indicating that the trap states are occupied by the charge carriers.^[^ ^46^, ^47^, ^48^ ^]^ The trap state density (*N*_t_) could be calculated from the trap‐filled limit voltage (*V*_TFL_) with the following equation: *N*_t_ = 2*εε*_0_ *V*_TFL_/*eL* ^2^, where *ε* and *ε*_0_ represent the average relative dielectric constants of CsCu_2_I_3_ and vacuum permittivity, respectively, *e* is the elementary electronic charge, and *L* is the thickness of films.^[^ ^46^ ^]^ By assuming that *ε* = 28.6,^[^ ^27^ ^]^ the *N*_t_ of pristine and PVK‐modified CsCu_2_I_3_ films were calculated to be 1.82 × 10^18^ and 7.19 × 10^17^ cm^‐3^, respectively (Figure 3f). The lower *N*_t_ suggests that the defects in CsCu_2_I_3_ films were effectively passivated after PVK introduction, which is in good agreement with the enhanced PLQY, improved films morphology, and the well‐optimized energy band alignment. Furthermore, the hole carrier mobility (*μ*_h_) of CsCu_2_I_3_ films with and without PVK modification is extracted by fitting the Mott–Gurney Law:^[^ ^38^ ^]^ *μ*_h_ = 8*JL* ^3^/9*εε*_0_ *V* ^2^, where *J* and *V* are the current density and applied voltage, respectively.^[^ ^46^ ^]^ The *μ*_h_ substantially increases from 5.47 × 10^‐8^ to 1.12 × 10^‐6^ cm^2^ V^‐1^ s^‐1^ after the CsCu_2_I_3_ films were passivated by PVK, indicating an improved hole injection and increased exciton source, which facilitates the carrier transportation and recombination under the electric field.^[^ ^37^ ^]^ Figure S10 (Supporting Information), presents a higher current density for electron‐only device than that of the hole‐only device based on the pristine CsCu_2_I_3_, suggesting that the electron dominates the injection process in pristine device, resulting in an imbalance of carrier injection. Thankfully, the current density of the hole‐only device based on PVK‐modified CsCu_2_I_3_ increased significantly, and thus a well‐balanced carrier injection was finally obtained.^[^ ^36^ ^]^ The polymer PVK with excellent hole transport properties permeates along the grain boundary into the CsCu_2_I_3_ films and connects with the HTLs at the bottom of the films well, which may be the reasons for enhanced *μ*_h_ and balanced carrier injection. Therefore, the PVK could effectively passivate the grain boundaries, endowing the films fewer grain boundary defects and higher hole injection efficiency simultaneously, thereby suppressing the excitons from being trapped by defects and increasing the probability of exciton recombination.^[^ ^38^, ^41^ ^]^ Thus, a substantial promotion of device performance can be expected.

**Figure 4:**
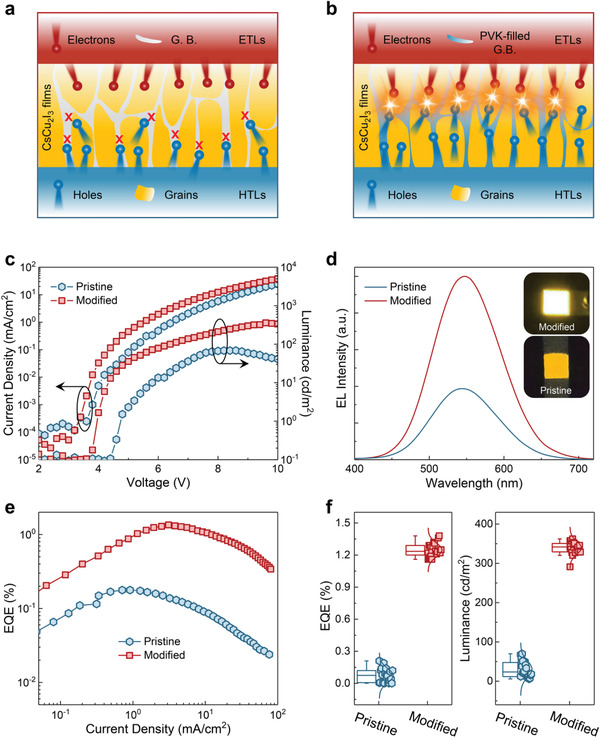
EL performances of the yellow LEDs with and without PVK modification. a,b) Schematic illustrations of the charge carrier injection and recombination in LEDs without (a) and with (b) PVK modification. c) Current density–voltage–luminance curves of the LEDs based on the pristine and PVK‐modified (0.2 mg mL^−1^) CsCu_2_I_3_ films. d) EL spectra of the pristine and PVK‐modified LEDs captured at 6.0 V. The insets show their corresponding photographs with a 4 mm^2^ emitting area. e) EQE versus current density of the pristine and PVK‐modified LEDs. f) Statistical distribution of the EQE and luminance of the pristine and PVK‐modified LEDs.

Based on the experimental and theoretical results discussed above, the possible mechanisms for defect passivation and hole injection improvement in CsCu_2_I_3_ films caused by PVK can be described by the schematic illustrations shown in **Figures** **4**a,b. Before the passivation, the defect states (such as Cu_Cs_ and Cu_I_ antisite defects) existed at the grain boundaries would trap the injected carriers with a very large probability,^[^ ^35^, ^37^ ^]^ which plays an adverse role in charge carrier transportation and recombination (Figure 4a). In contrast, when the grain boundary areas are penetrated by PVK additives, these defects are effectively passivated, and the penetrative areas can act as the hole injection paths for carrier transportation (Figure 4b). In this case, all available crystal grains are more exposed to the hole‐transporter, which can partly offset the disadvantages of poor hole injection and transport. Moreover, the embedded structure of CsCu_2_I_3_/PVK can spatially confine the nondirectional diffusion of holes and electrons,^[^ ^36^, ^37^ ^]^ which is also responsible for their enhanced radiative recombination.

After the PVK passivation mechanisms were clarified, we proceed to assess the device performance of the complete LEDs. Figure 4c shows the current density–voltage–luminance curves of the pristine and PVK‐modified LEDs (0.2 mg mL^−1^). One can see that the modified LED exhibit a lower turn‐on voltage compared with the pristine device, which benefits from the enhanced hole injection and reduced injection barrier between HTLs and emitters.^[^ ^43^ ^]^ In addition, the luminance of the modified LED was substantially improved, with the maximum value increasing from 68.4 to 357.8 cd m^−2^, producing an enhancement ratio of ≈423%. Meanwhile, a premature luminance roll‐off occurs at ≈8.0 V for the pristine device, reflecting an undesirable energy loss from the unbalanced carrier injection.^[^ ^37^ ^]^ Figure 4d presents the EL spectra of the pristine and PVK‐modified LEDs captured at 6.0 V, and the insets display the corresponding operating photographs of an emitting unit with an area of 4 mm^2^. One can observe that both devices show similar broadband yellow emission with a wide spectra coverage range of 400–720 nm, corresponding to the Commission Internationale de l'Eclairage color coordinates of (0.43, 0.53), making them more promising for healthy lighting applications without blue‐light hazard.^[^ ^49^, ^50^, ^51^ ^]^ Notably, the modified LED demonstrates an obvious enhancement in EL intensity compared to the pristine device, consistent with their significantly different brightness visible to the naked eyes. Besides, the EQE of two devices were measured and plotted in Figure 4e as a function of bias voltage. At all comparable biases, the modified device exhibits higher values. Typically, at a current density of ≈3.1 mA cm^−2^, the modified device reaches up a peak EQE of 1.35%, about 8.5‐fold in comparison with the pristine device (0.16%). The detailed device performances of two devices are summarized in Table S3 (Supporting Information). Such significantly improved device performances can be attributed to the combined effects of high‐quality CsCu_2_I_3_ films with less defect states, improved carrier injection balance, and efficient radiative recombination of carriers through the PVK modification.^[^ ^45^ ^]^ To assess the reproducibility of the studied LEDs, twenty devices with and without PVK modification were measured under the respective optimal conditions. As shown in Figure 4f, the average EQE of 0.10% and 1.23% as well as the average luminance of 23.3 and 341.2 cd m^−2^ are obtained from the statistics results for two devices, manifesting a good reproducibility of the device performances associated with the use of PVK.

**Figure 5:**
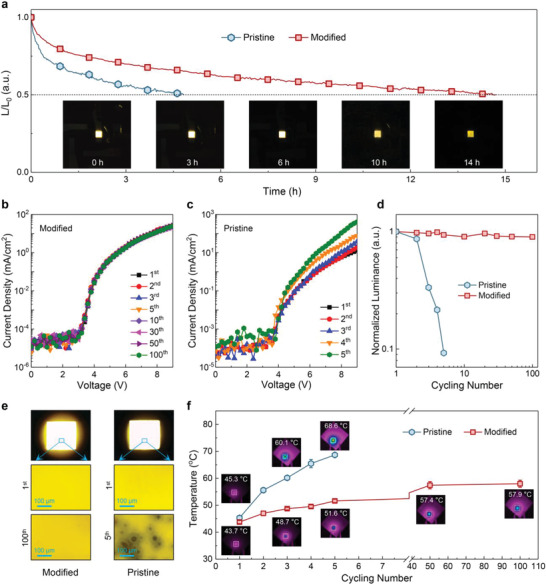
Stability study of the yellow LEDs. a) *T*_50_ measurement of the pristine and PVK‐modified LEDs. The insets present the corresponding photographs of the PVK‐modified LEDs after different running periods. b,c) Current density–voltage curves of the PVK‐modified (b) and pristine (c) LEDs with multiple scan cycles. The scanning range is 0−9.0 V and the scanning rate is 0.2 V per step for all measurements. d) Evolution of the luminance according to the number of scan cycles. e) Optical microscope images of the pristine and PVK‐modified devices at different scan cycles. f) Surface temperature evolution of two devices measured after each scan cycle aging.

Stability of LEDs is of crucial important for their practical applications, so we measured the operational lifetime of the fabricated LEDs by monitoring the luminance decay under continuous operation in ambient conditions.^[^ ^52^ ^]^ **Figure** **5**a shows the luminance of the pristine and modified LEDs versus operating time at a constant current density of ≈3 mA cm^−2^, and a visible difference was observed. The pristine device shows a rapid emission degradation with a short half‐lifetime (*T*_50_,^[^ ^42^ ^]^ luminance decay to half of its initial value) of only ≈4.6 h. In contrast, the modified device exhibits a substantially improved stability, delivering an extended *T*_50_ of ≈14.6 h at an initial luminance of ≈100 cd m^−2^, which is greatly superior to other previous studies on yellow LEDs based on perovskite systems (Table S4, Supporting Information). Figure S11 (Supporting Information), shows the corresponding evolution of EL spectra of the pristine and modified devices during the whole operation tracking, and no changes on spectral shape and peak position were observed. Moreover, the storage stability of the pristine and modified LEDs stored in ambient conditions (25 °C, 40–60% humidity) was also evaluated. As shown in Figure S12 (Supporting Information), the EQE of the modified device remains about 60% of its initial value after 30‐day storage, while the pristine device loses 75% of the initial performance in just six days, testing under the same conditions. Herein, we attribute the significantly enhanced stability of modified device to the following three key points. First, the PVK‐modified CsCu_2_I_3_ films are characterized by a more compact morphology, providing less a chance for the emitter being exposed to the moisture and oxygen,^[^ ^53^ ^]^ thereby stabilizing the device operation. The improved moisture stability for the PVK‐modified CsCu_2_I_3_ films confirms the above statement, as evidenced in Figure S13 (Supporting Information). Second, the defect states at the grain boundaries in CsCu_2_I_3_ films were effectively passivated by the polymer PVK, thus the defect‐mediated nonradiative process, which then accounts for the heating issue, can be inhibited. Even the device was operated for a long time, the local Joule heating is not obvious, which will be discussed later in detail. Third, the notably enhanced charge injection/transport capability caused by the PVK modification is also an important factor for the stable running of the device. In theory, the boosted electrical properties of the emitter would balance the spatial distribution of injection holes and electrons and alleviate the undesired carrier accumulation at the trap sites, leading to facilitated device operation.^[^ ^54^, ^55^ ^]^

We finally examined the durability of the studied LEDs through a cycle switching test. Both pristine and modified LEDs were measured by multiple scans from 0 to 9.0 V with a scanning rate of 0.2 V per step.^[^ ^42^ ^]^ As shown in Figure 5b–d, the current density and luminance of the modified LED remain nearly unchanged upon 100 cycles of forward voltage driving, showing a reliable repeated stability under electrical stress. In sharp contrast, the current density of the pristine LED exhibits a significant rise with scan cycles, and the corresponding luminance drops by more than 90% within only 5 cycles of voltage scan, as shown in Figures 5c,d. The luminance degradation of the pristine device under multiple electrical stress can be more intuitively presented by the optical microscope images. As presented in Figure 5e, the yellow emission of the modified LED spreads evenly across the entire emission area after switching aging. While, a large amount of emission dark spots with an average size of ≈25 µm appear in the pristine device even with a much shorter scan cycles, showing a poor durability. The above observations are consistent with the results of transient PL decay of two devices (Figure S14, Supporting Information). Taking into account the existence of pinholes in the pristine CsCu_2_I_3_ films, there is likely to be a high current filament through the pinholes, where local Joule heat generates and accumulates. Thus, the resulting LED cannot survive well after undergoing multiple scan cycles.^[^ ^56^ ^]^ For further confirmation, the surface temperature of the pristine device after each scan cycle aging was monitored. As shown in Figure 5f, an obvious temperature increase from 45.3 °C (first) to 68.6 °C (fifth) was observed with increasing the scan cycles, producing a temperature sensitivity coefficient of ≈4.66 °C per cycle. In contrast, the highest surface temperature of the modified device only reaches about 57.9 °C even after 100 scan cycles, with the temperature sensitivity coefficient as low as ≈0.142 °C per cycle. Considering that the accumulated heat would give rise to a rapid proliferation of structural defects, producing added nonradiative recombination centers in CsCu_2_I_3_ emitter. Thus it seems reasonable that the radiative recombination probability of injected carriers is reduced rapidly with scan cycle aging for the pristine device. The above results strongly confirm that the introduction of PVK in CsCu_2_I_3_ films could suppress effectively the nonradiative recombination by passivating the defect states, resulting in mitigated Joule heating effect and improved device repeated durability.

## 3 Conclusion

In summary, we have successfully demonstrated an eco‐friendly and stable yellow LED with the PVK‐modified CsCu_2_I_3_ films as the single emitter. Experimental and theoretical results show that the incorporation of polymer PVK into the CsCu_2_I_3_ films can effectively passivate the grain boundary defects by interacting with the uncoordinated Cu^+^, suppressing the formation of Cu_Cs_ and Cu_I_ anti‐site defects. Besides, the reduced grain size of CsCu_2_I_3_ films after PVK modification enables an enhanced spatial confinement and decreased diffusion length of excitons, which supports a prolonged PL lifetime and an increased PLQY. Simultaneously, the hole‐transporting PVK could effectively modulate the electrical properties of CsCu_2_I_3_ films and the resulting device, leading to enhanced hole injection/transport, favorable energy band alignment, and efficient carrier recombination. Benefited from these synergistic effects, the yellow LEDs with PVK modification reach a peak EQE of 1.35%, about 8.5‐fold in comparison with the pristine device (0.16%). Moreover, the PVK‐modified device demonstrated a robust operational stability in air ambient, producing a record *T*_50_ of 14.6 h among the perovskites‐based yellow LEDs. These findings evince that the synergistic effect of defect passivation and electrical property regulation by polymer PVK provides a novel promising strategy for fabricating high‐performance eco‐friendly yellow LEDs, suggesting added potentials in next‐generation lighting technologies.

## 4 Experimental Section

### Materials

Cesium iodide (CsI, 99.9%) and copper iodide (CuI, 99.999%) were purchased from Sigma‐Aldrich. N,N‐dimethyl‐formamide (DMF, 99.9%), toluene (99%), and Dimethyl sulfoxide (DMSO) were purchased from Beijing Chemical Reagent Co., Ltd., China. Poly(ethylenedioxythiophene):polystyrenesulfonate (PEDOT:PSS), poly(9‐vinlycarbazole) (PVK), poly[N,N′‐bis(4‐butylphenyl)‐N,N′‐bisphenylbenzidine] (Poly‐TPD), poly[(9,9‐dioctylfluor‐enyl‐2,7‐diyl)‐co‐(4,4″‐(N‐(p‐butylphenyl))diphenylamine)] (TFB), 4,4′,4″‐tris(carbazol‐9‐yl) triphenylamine (TCTA), 1,3,5‐tri (m‐pyrid‐3‐yl‐phenyl)benzene (TmPyPB), and lithium fluoride (LiF, 99.99%) were purchased from Xi'an Polymer Light Technology Corp. All these chemical materials were directly used without further purification.

### Preparation of the CsCu_2_I_3_ Films

The precursor solutions were prepared by mixing 0.5 m CsI and 0.5 m CuI in a miature of DMF and DMSO (1:1 v/v) with different amounts of PVK (0, 0.1, 0.2, 0.3 mg mL^−1^) and stirring constantly at 70 °C for 12 h. After that, a dark red precursor solution was obtained by filtering the crude solution through the PTFE 0.22 µm filters in argon‐filled glovebox. For films fabrication, the presursor solution was spin‐coated on the quartz substrates with a low‐speed spinning (500 rpm, 5 s) and a high‐speed spinning (3000 rpm, 60 s). During the spin‐coating process, 100 µL toluene was dropped onto the top of the spinning layer in 45 s to assist the crystallization process. Then the sample was transferred to a hot plate for annealing treatment at 100 °C for 40 min.

### Device Fabrication

The patterned ITO‐coated glass substrates with emitting areas of 4 mm^2^ were sequentially cleaned by sonication in acetone, ethyl alcohol, and deionized water for 15 min, respectively, and were further treated by ultraviolet ozone illumination for 20 min. Then, the PEDOT:PSS aqueous solution was spin‐coated onto the ITO substrate at 4000 rmp for 60 s and baked in air ambient at 130 °C for 20 min. Following that, the substrates were transferred into argon‐filled glove box, and Poly‐TPD solutions (6 mg mL^−1^ in chlorobenzene) and PVK solutions (6 mg mL^−1^ in chlorobenzene) were spin‐coated onto the PEDOT:PSS layers in turn at 4000 rpm for 60 s and baked at 120 °C for 30 min. The emissive layers with different PVK contents were spin‐coated on the substrate through the method described above. Finally, TmPyPB, LiF, and metal Al layers were thermally evaporated with the thicknesses of 55, 1, and 100 nm, respectively, in a thermal evaporation chamber under a high vacuum of ≈1 × 10^−4^ Pa.

### Materials and Device Characterizations

The morphologies and surface roughness of the CsCu_2_I_3_ films were measured by SEM (Jeol‐7500F, 15 keV) and AFM (Dimension Icon, Bruker Corporation), respectively. The crystallinity characterizations of CsCu_2_I_3_ films were analyzed by XRD (Panalytical X'Pert Pro). The depth profile XPS determinations were conducted by a SPECS XR50 system equipped with an Ar gas cluster ion beam gun (Mg K*α* source, 1253.6 eV). The steady‐state PL and absorption spectra of the products were tested using a steady‐state PL spectrum (Horiba; Fluorolog‐3) and Shimadzu UV‐3150 spectrophotometer, respectively. Transient‐state PL measurements were performed using a fluorescence lifetime measurement system with a pulsed nano‐LED (Horiba; 340 nm). The absolute PLQY of the samples was measured by using a fluorescence spectrometer (Horiba; FluoroMax‐4) with an integrated sphere (Horiba; Quanta‐*φ*). The current–voltage–luminance curves of device were measured using a Keithley 2400 source meter coupled with a PR705 SpectraScan spectrophotometer (Photo Research). The EL spectra were collected using a spectra acquisition system including lock‐in amplifier (Stanford; SR830‐DSP) and photomultiplier tube (PMTH‐S1‐R1527). The EQE of device was derived by recording the light output power of the device by using a digital optical power meter (THORLABS, PM100D) and a silicon photodetector (THORLABS, S120VC). Under continuous current operation provided by a Keithley 2400 source, the half‐lifetime (*T*_50_) of devices was tested.

### Theoretical Calculations

The structural optimizations and electronic structure calculations are performed based on density functional theory (DFT) as implemented in the Vienna Ab Initio Simulation Package (VASP) code, based on the projector‐augmented‐wave (PAW) method with a cutoff energy of 600 eV.^[^ ^57^ ^]^ All of configurations of CsCu_2_I_3_ based materials were fully optimized. The generalized gradient form (GGA) of the exchange‐correlation functional (Perdew–Burke–Ernzerhof 96, PBE) was adopted.^[^ ^58^ ^]^ A revised Perdew–Burke–Ernzerhof generalized gradient approximation (PBEsol) was used for the exchange‐correlation. PBEsol functional has been introduced to improve the equilibrium properties of solids.^[^ ^59^ ^]^ Valence–core interactions were described by PAW pseudopotentials. The Brillouin zone sampling is carried out using the (3 × 3 × 3) Monkhorst‐Pack grids for surface and Gamma for the structure.^[^ ^57^ ^]^ The convergence tolerance of energy is 1×10^−6^ eV, maximum force is 0.001 eV Å^−1^, and maximum displacement is 0.001 Å. The band structure and density of states are corrected using HSE06, and the convergence parameters such as K‐point and cut‐off energy are the same as PBE. The formation energy of defects was defined as: *E*_f_ = *E*_a_ + *E*_b_ − *E*_pristine_. And the formation energy of defects passivated by PVK was defined as: *E*_f_ = *E*_a_ + *E*_c_ − *E*_passivated_, where *E*_a_ and *E*_pristine_ are the total energy of CsCu_2_I_3_ with and without defects, respectively, and *E*_passivated_ is the total energy of CsCu_2_I_3_ after passivation. *E*_b_ is the chemical potential of the corresponding ions (Cu_Cs_, Cu_I_, Cu_int._ V_Cu_) and *E*_c_ is the chemical potential of the passivated ions.

## Conflict of Interest

The authors declare no conflict of interest.

## Supporting information

Supporting Information

## Data Availability

The data that support the findings of this study are available in the supplementary material of this article.
