# Supplementary material for: Carbazole‐Containing Polymer‐Assisted Trap Passivation and Hole‐Injection Promotion for Efficient and Stable CsCu2I3‐Based Yellow LEDs
Source: Adv Sci (Weinh). 2022 Jul 3;9(27):2202408. doi: 10.1002/advs.202202408 (PMC9507358; doi:10.1002/advs.202202408)
Supplement: Supplementary file 1 — Supporting Information [file ADVS-9-2202408-s001.pdf]

ADVANCED SCIENCE
Open Access

Supporting Information

for *Adv. Sci.*, DOI 10.1002/advs.202202408

Carbazole-Containing Polymer-Assisted Trap Passivation and Hole-Injection Promotion for Efficient and Stable $CsCu_2I_3$-Based Yellow LEDs

Zhuangzhuang Ma, Xinzhen Ji, Meng Wang, Fei Zhang, Zibin Liu, Dongwen Yang, Mochen Jia, Xu Chen, Di Wu, Yu Zhang, Xinjian Li, Zhifeng Shi and Chongxin Shan**

Copyright WILEY-VCH Verlag GmbH & Co. KGaA, 69469 Weinheim, Germany, 2022.

Supporting Information

Carbazole-Containing Polymer-Assisted Trap Passivation and Hole-Injection Promotion for Efficient and Stable $CsCu_2I_3$-Based Yellow LEDs

Zhuangzhuang Ma, Xinzhen Ji, Meng Wang, Fei Zhang, Zibin Liu, Dongwen Yang, Mochen Jia, Xu Chen, Di Wu, Yu Zhang, Xinjian Li, Zhifeng Shi, and Chongxin Shan**

Dr. Zhuangzhuang Ma, Dr. Xinzhen Ji, Dr. Meng Wang, Dr. Fei Zhang, Dr. Zibin Liu, Dr. Dongwen Yang, Dr. Mochen Jia, Dr. Xu Chen, Dr. Di Wu, Prof. Xinjian Li, Prof. Zhifeng Shi, Prof. Chongxin Shan
Key Laboratory of Materials Physics of Ministry of Education, School of Physics and Microelectronics, Zhengzhou University, Daxue Road 75, Zhengzhou 450052, China
E-mail: shizf@zzu.edu.cn; cxshan@zzu.edu.cn
Prof. Yu Zhang
State Key Laboratory on Integrated Optoelectronics, College of Electronic Science and Engineering, Jilin University, Qianjin Street 2699, Changchun 130012, China

Keywords: yellow light-emitting diodes, copper halide, defect passivation, charge carrier injection, stability

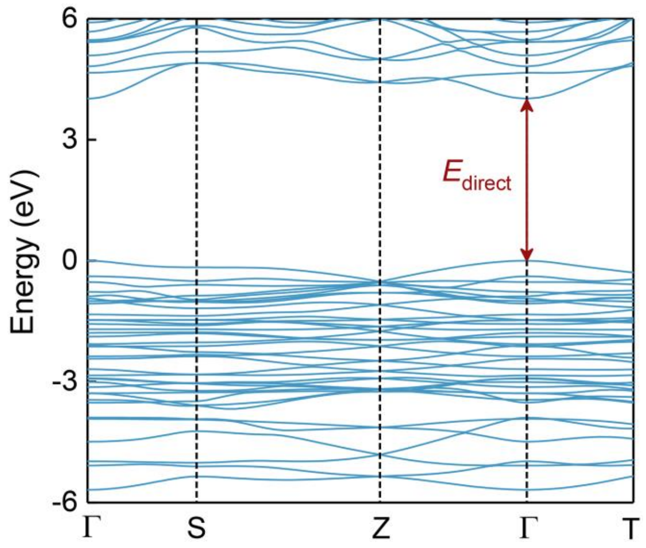

Figure S1. The calculated band structure of $CsCu_2I_3$.

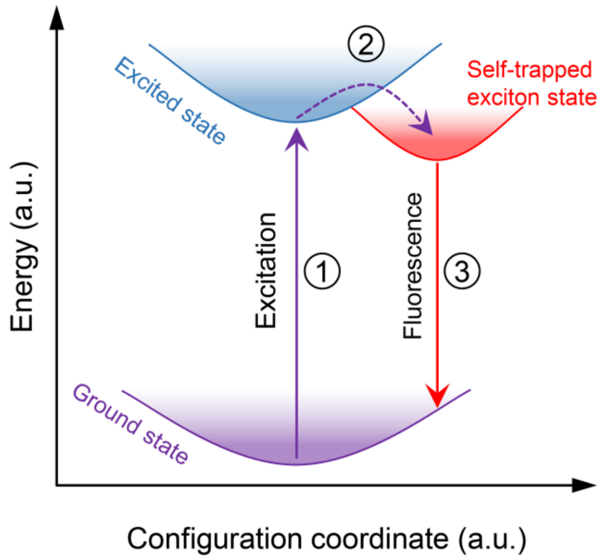

Figure S2. Configuration coordinate diagram for the self-trapped exciton emission mechanism of $CsCu_2I_3$.

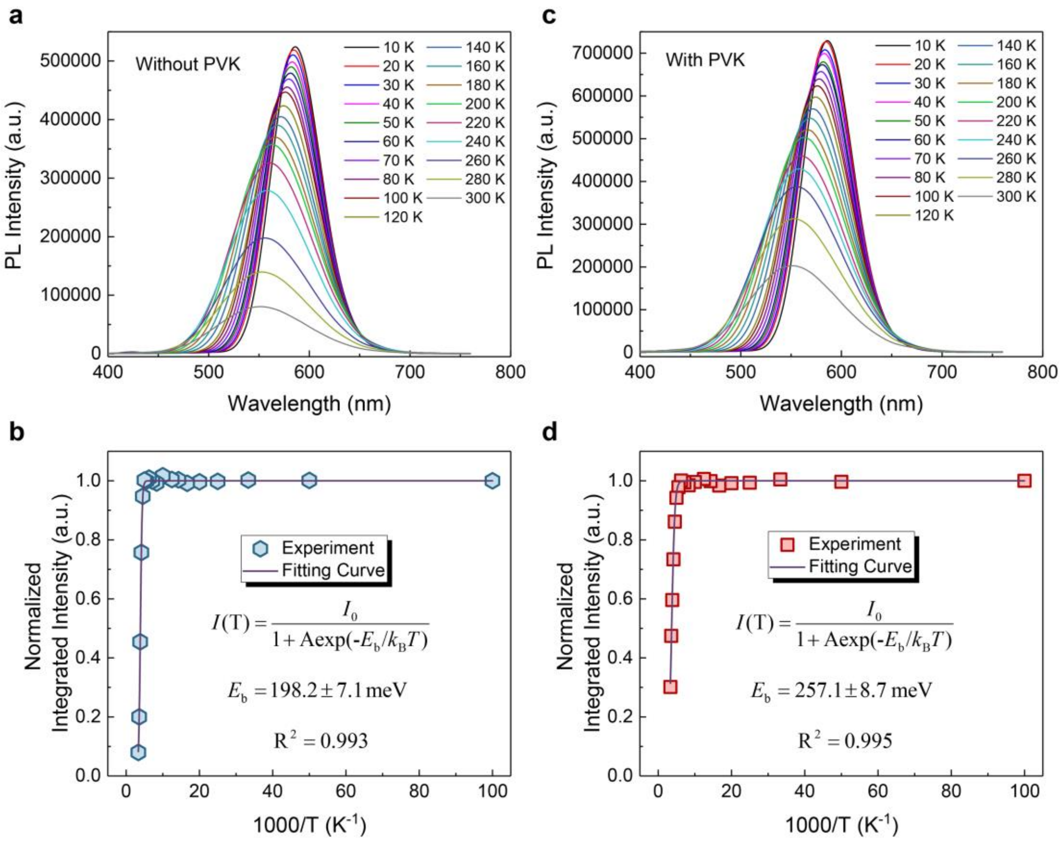

Figure S3. a, c) Pseudocolor map of temperature-dependent PL spectra of the $CsCu_2I_3$ films without and with PVK modification. b, d) Integrated PL intensity of the $CsCu_2I_3$ films without and with PVK modification as a function of reciprocal temperature.

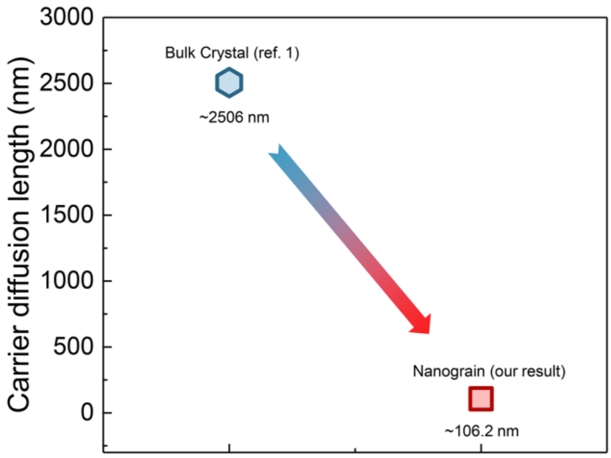

Figure S4. Exciton diffusion length of $CsCu_2I_3$ films and bulk crystals. The exciton diffusion length (L_D) could be calculated according to the following equation[1–3]: $L_D = \sqrt{<\tau_{ave.}> \mu k_B T / e}$, where $<\tau_{ave.}>$ is the average lifetime, μ is the carrier mobility, k_B is the Boltzmann constant, and e is the elementary charge. The L_D of $CsCu_2I_3$ bulk crystal comes from ref. 1.

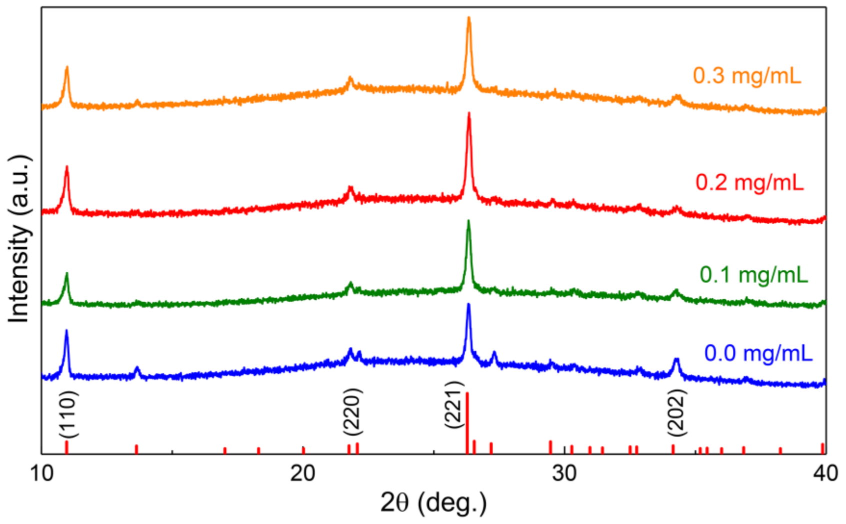

Figure S5. XRD patterns of the $CsCu_2I_3$ films modified with different PVK concentrations.

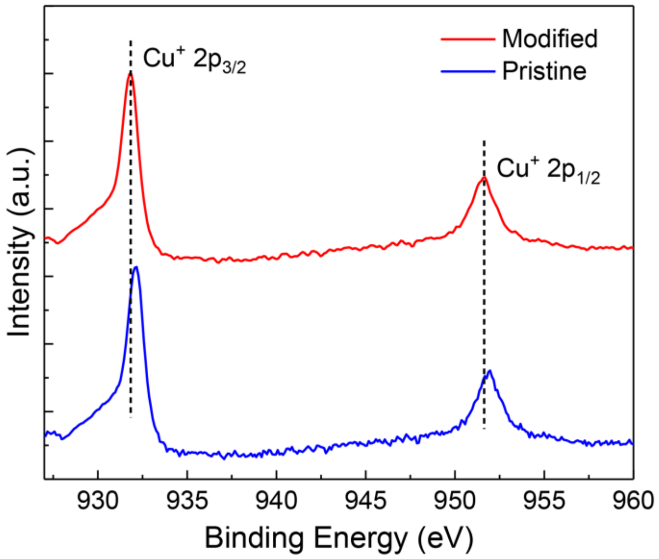

Figure S6. XPS spectra of Cu 2p peaks of the pristine and PVK-modified $CsCu_2I_3$ films.

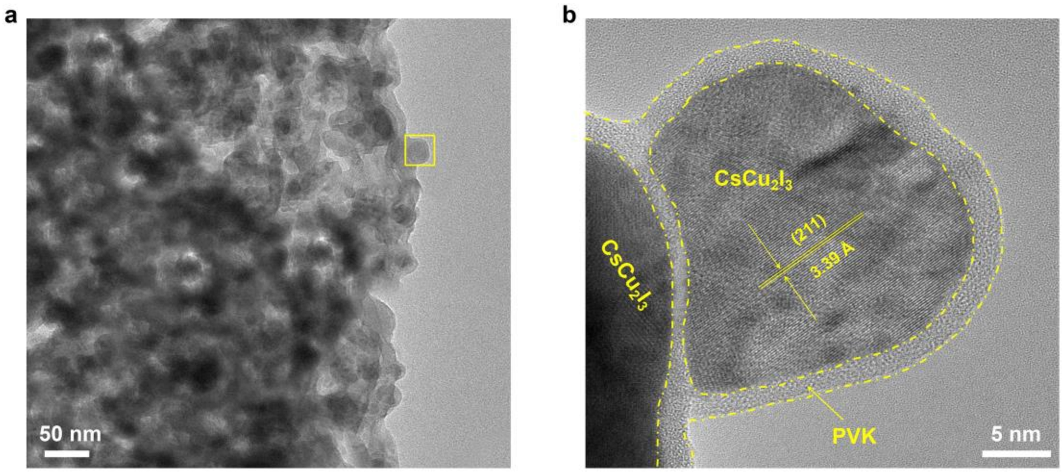

Figure S7. a) Low-magnification TEM image of the PVK-modified $CsCu_2I_3$ films. b) High-resolution TEM image of the PVK-modified $CsCu_2I_3$ films, showing the presence of PVK wall among the adjacent $CsCu_2I_3$ grains.

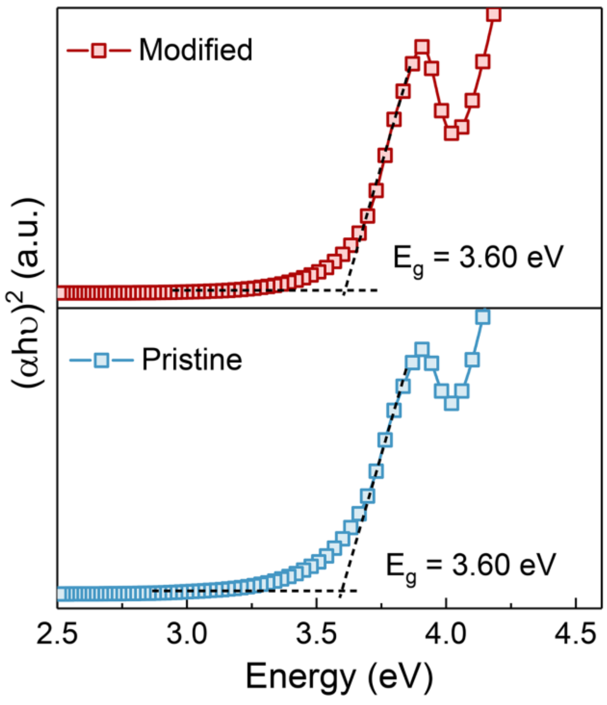

Figure S8. Tauc plots of the pristine and PVK-modified $CsCu_2I_3$ films.

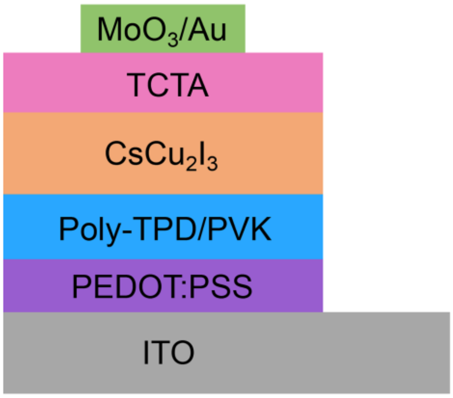

Figure S9. Hole-only devices with the structure of ITO/PEDOT:PSS/Poly-TPD/PVK/$CsCu_2I_3$/TCTA/MoO_3/Au based on the pristine and PVK-modified $CsCu_2I_3$ films.

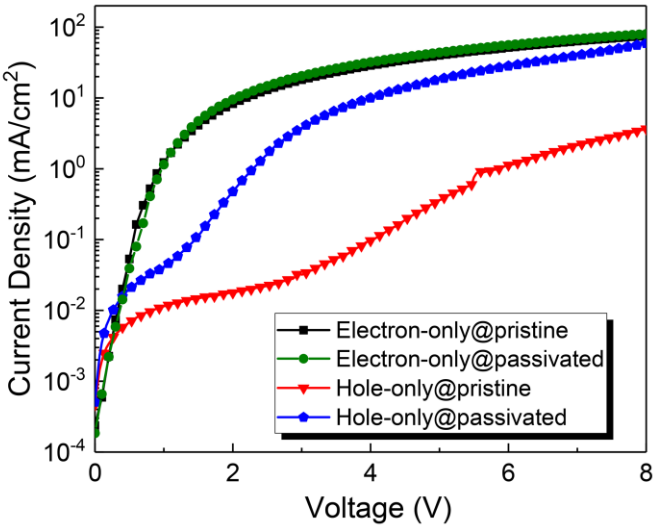

Figure S10. Current density–voltage curves of the electron-only devices (ITO/TmPyPB/$CsCu_2I_3$/TmPyPB/LiF/Al) and hole-only devices (ITO/PEDOT:PSS/Poly-TPD/PVK/$CsCu_2I_3$/TCTA/MoO_3/Au) based on the pristine and PVK-modified $CsCu_2I_3$ films.

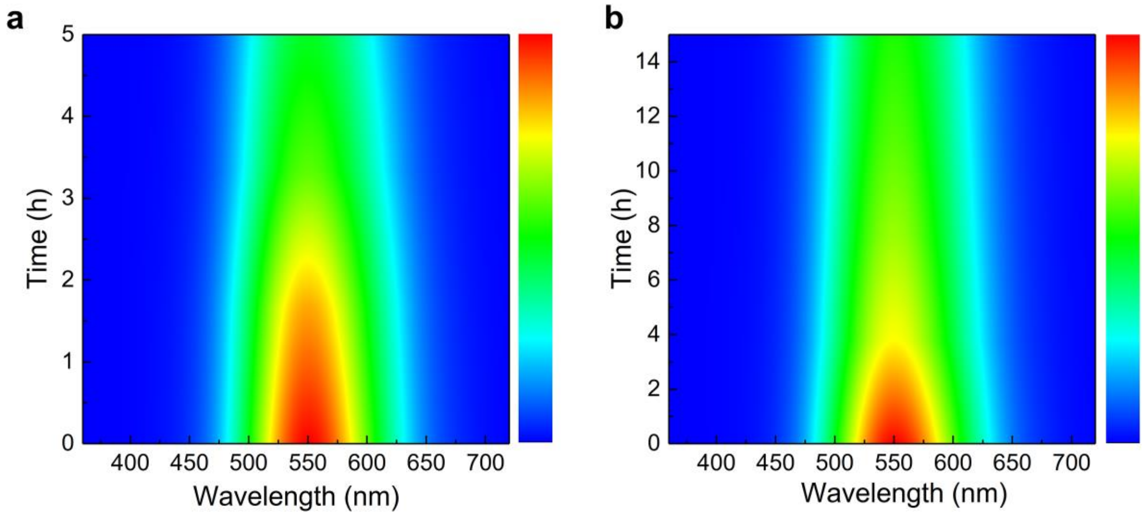

Figure S11. EL spectra as a function of operational time for pristine a), and PVK-modified b) devices.

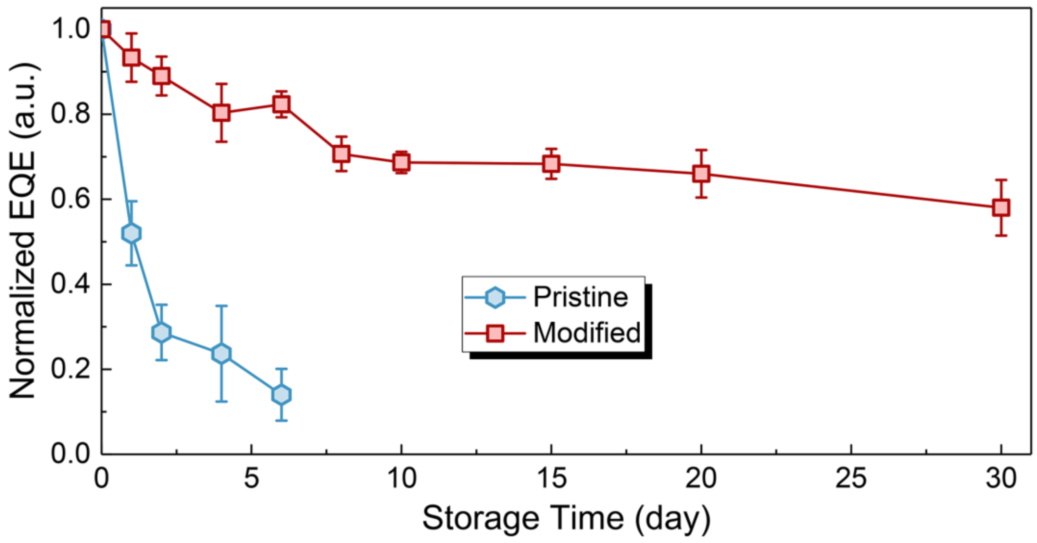

Figure S12. EQE evolution of the pristine and PVK-modified LEDs after different storage time in ambient conditions (25 °C, 30–50% humidity).

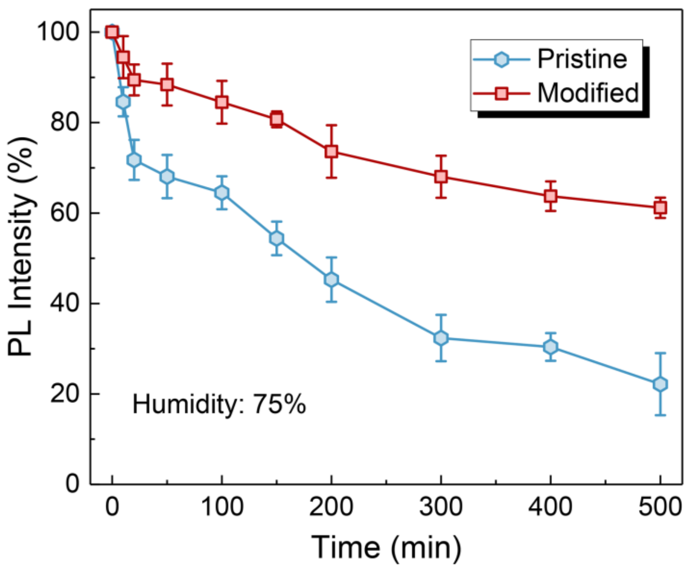

Figure S13. PL intensity evolution of the $CsCu_2I_3$ films with and without PVK modification under moisture aging.

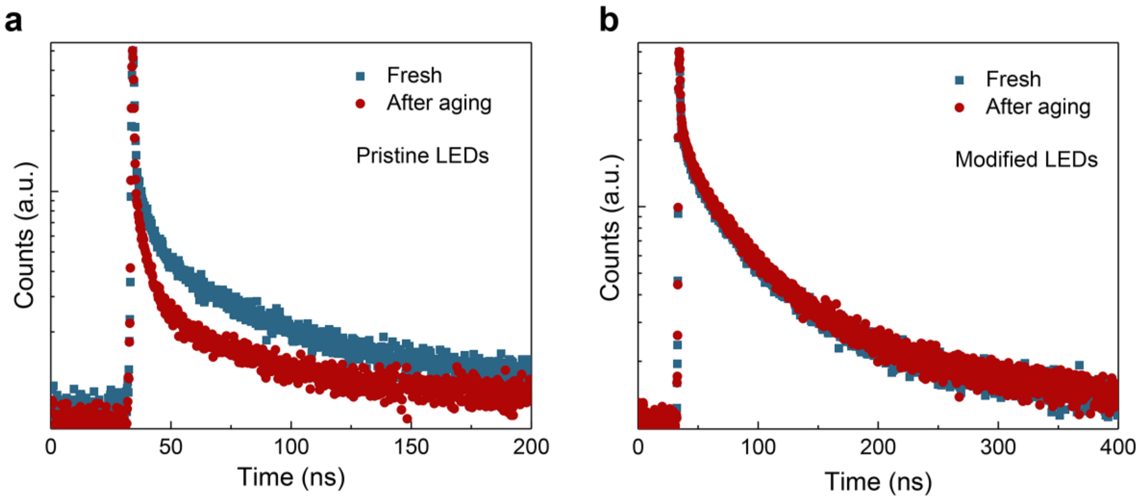

Figure S14. Transient PL decay curves of the pristine a), and PVK-modified b) devices before and after switching aging.

Table S1. Fitting parameters of the time-resolved PL decay curves for $CsCu_2I_3$ films with different concentrations of PVK modification

Samples	K_1 (%)	τ_1 (ns)	K_2 (%)	τ_2 (ns)	$\tau_{ave.}$ (ns)
0 mg/mL	80.2	1.84	19.8	28.43	7.1
0.1 mg/mL	72.6	2.04	27.4	35.89	11.3
0.2 mg/mL	60.8	3.05	39.2	49.85	21.4
0.3 mg/mL	65.4	2.66	34.6	41.77	16.2

$\tau_{ave.} = (K_1 \times \tau_1^2 + K_2 \times \tau_2^2)/(K_1 \times \tau_1 + K_2 \times \tau_2)$

Table S2. Formation energy of Cu-related defects in $CsCu_2I_3$ under Cu poor conditions without and with PVK modification

	Cu_{Cs} (eV)	Cu_I (eV)	$Cu_{int.}$ (eV)	V_{Cu} (eV)
$CsCu_2I_3$	1.715	2.219	2.351	1.983
$CsCu_2I_3$-PVK	1.981	2.440	2.359	2.003

Table S3. Summary of the device performances of the studied LEDs with and without PVK modification

Emitters	Turn-on voltage (V)	EL peak (nm)	Max. luminance (cd/m^2)	Max. EQE (%)	Max. current efficiency (cd/A)
Without PVK	4.4	550	68.4	0.16	0.71
With PVK	3.8	550	357.8	1.35	5.45

Table S4. Summary of the EL performance of reported yellow LEDs in literatures

Emitter materials	Lead-free (Yes/No)	Peak EQE (%)	Initial luminance (cd/m^2)	Lifetime (T_{50})	Ref.
$CsPbBr_2I$	No	3.7	100	146 min (T_{80})	4
$CsPb(Br_{0.55}I_{0.45})_3$	No	–	–	40 min	5
$CsPbBr_{3-x}I_x$	No	0.13	~100	5 min	6
$(BIZ)_2Mn_{0.23}Pb_{0.77}I_4$	No	0.045	–	–	7
$Cs_2(Ag_{0.6}Na_{0.4})InCl_6$	Yes	–	~30	10 min	8
$CsCu_2I_3$	Yes	~0.15	–	–	9
$CsCu_2I_3$	Yes	0.17	~10	310 min	10
$CsCu_2I_3$	Yes	1.35	~100	876 min	This work

References

[1] X. Mo, T. Li, F. Huang, Z. Li, Y. Zhou, T. Lin, Y. Ouyang, X. Tao, C. Pan, *Nano Energy* **2021**, *81*, 105570.

[2] G. R. Yettapu, D. Talukdar, S. Sarkar, A. Swarnkar, A. Nag, P. Ghosh, P. Mandal, *Nano Lett.* **2016**, *16*, 4838.

[3] H. Cho, S.-H. Jeong, M.-H. Park, Y.-H. Kim, C. Wolf, C.-L. Lee, J. H. Heo, A. Sadhanala, N. Myoung, S. Yoo, S. H. Im, R. H. Friend, T.-W. Lee, *Science* **2015**, *350*, 1222.

[4] J. Li, L. Yang, Q. Guo, P. Du, L. Wang, X. Zhao, N. Liu, X. Yang, J. Luo, J. Tang, *Sci. Bull.* **2021**, *67*, 178.

[5] Y. He, J. Gong, Y. Zhu, Feng. Y, H. Peng, W. Wang, H. He, H. Liu, L. Wang, *Opt. Mater.* **2018**, *80*, 1.

[6] P. Vashishtha, J. Halpert, *Chem. Mater.* **2017**, *29*, 5965.

[7] L. Zhang, T. Jiang, C. Yi, J. Wu, X. K. Liu, Y. He, Y. Miao, Y. Zhang, H. Zhang, X. Xie, P. Wang, R. Li, F. Gao, W. Huang, J. Wang, *J. Phys. Chem. Lett.* **2019**, *10*, 3171.

[8] J. Luo, X. Wang, S. Li, J. Liu, Y. Guo, G. Niu, L. Yao, Y. Fu, L. Gao, Q. Dong, C. Zhao, M. Leng, F. Ma, W. Liang, L. Wang, S. Jin, J. Han, L. Zhang, J. Etheridge, J. Wang, Y. Yan, E. H. Sargent, J. Tang, *Nature* **2018**, *563*, 541.

[9] R. Roccanova, A. Yangui, G. Seo, T. D. Creason, Y. Wu, D. Y. Kim, M.-H. Du, B. Saparov, *ACS Mater. Lett.* **2019**, *1*, 459.

[10] Z. Ma, Z. Shi, C. Qin, M. Cui, D. Yang, X. Wang, L. Wang, X. Ji, X. Chen, J. Sun, D. Wu, Y. Zhang, X. Li, L. Zhang, C. Shan, *ACS Nano* **2020**, *14* ,4475.
